# Gut microbiota–derived tryptophan metabolites: molecular mechanisms, nutritional strategies and implications for swine health

**DOI:** 10.3389/fvets.2026.1807477

**Published:** 2026-05-15

**Authors:** Yang Wen

**Affiliations:** National Key Laboratory of Pig Genetic Improvement and Germplasm Innovation, Jiangxi Agricultural University, Nanchang, China

**Keywords:** gut microbiota, immunometabolism, indole derivatives, probiotic, swine, tryptophan metabolism

## Abstract

Tryptophan, an essential amino acid, has nutritional value. Beyond that, it is an important signaling molecule that connects the gut microbiota with host physiology. While host-mediated pathways are well-characterized, the microbiota-driven indole pathway has emerged as a major modulator of host homeostasis. Commensal bacteria metabolize unabsorbed tryptophan into indole-3-propionic acid (IPA), indole-3-lactic acid (ILA) and other bioactive indole derivatives. These bioactive indole derivatives can act as ligands for aryl hydrocarbon receptor (AhR) and pregnane X receptor (PXR). This review makes a summary of the main tryptophan metabolic pathways. It also elucidates the molecular mechanisms by which microbial metabolites derived from tryptophan restore the integrity of the intestinal barrier, maintain immune homeostasis, and modulate host metabolism. Building on this, we discuss nutritional strategies, such as dietary patterns and probiotic interventions, and their potential to modulate tryptophan metabolism. Using pigs as a translational model, we summarize the potential applications of these metabolites in alleviating weaning stress and improving growth performance. This review focuses on tryptophan metabolism and provides a theoretical basis for microbiome interventions and precision nutrition strategies in swine production.

## 1 Introduction

As a metabolic organ, the gut microbiome generates a variety of small-molecule metabolites via *de novo* synthesis and from dietary substrates (1). These metabolites exert an influence on nutrient absorption (2), energy metabolism (3) and immune system regulation (4). Additionally, they contribute to colonization resistance (5). In terms of origin, they can be roughly divided into three types: microbially synthesized compounds (e.g., vitamins and neurotransmitter-like molecules), host-derived molecules modified by microbes (e.g., secondary bile acids), and metabolites produced from dietary components [e.g., short-chain fatty acids (SCFAs) and amino acid derivatives] (6). Among dietary-derived metabolites, indole derivatives generated from tryptophan metabolism represent key signaling molecules linking microbial metabolism to host physiological phenotypes. They get involved in regulating immune homeostasis, metabolic processes and intestinal barrier function (7).

Tryptophan is an aromatic amino acid that has an indole ring. It must be acquired from the diet (8). In the gastrointestinal tract, tryptophan follows three main metabolic routes: the host-mediated kynurenine and serotonin pathways, and the microbiota-driven indole pathway (9, 10). Commensal bacteria, including *Lactobacillus, Clostridium, Escherichia coli*, and *Bifidobacterium* species, express tryptophan-metabolizing enzymes to convert unabsorbed luminal tryptophan into indole and derivatives such as indole-3-acetic acid (IAA), indole-3-propionic acid (IPA), and indole-3-aldehyde (IAld) (11). Multiple indole compounds can function as ligands for aryl hydrocarbon receptor (AhR) or pregnane X receptor (PXR) (12–15). Animal studies demonstrate that indole can improve the integrity of the epithelial barrier and modulate intestinal inflammation (16). In addition, indole-3-lactic acid (ILA) and IAld can activate AhR and have been linked to immunoregulatory effects (7). IAA has been reported to influence hepatic lipid metabolism, suggesting that microbiota-derived indole derivatives may also regulate systemic metabolic phenotypes (17).

This review outlines the major metabolic routes of tryptophan with an emphasis on microbial pathways that generate indole derivatives. And it synthesizes mechanistic evidence connecting these metabolites to immune modulation, host metabolism and intestinal barrier integrity. Tryptophan metabolites are subject to the direct or indirect regulation of the gut microbial community, and thus we further discuss the potential of dietary and probiotic interventions that target microbial composition and function to modulate tryptophan metabolism. Finally, we focus on pigs as a translational model and summarize evidences linking microbial tryptophan-derived metabolites to improve intestinal health and growth performance. We also discuss the adverse effect of skatole on pork quality in swine production, aiming to inform the development of precision nutrition strategies for livestock.

## 2 Sources, absorption, and metabolism of tryptophan

**Figure 1:**
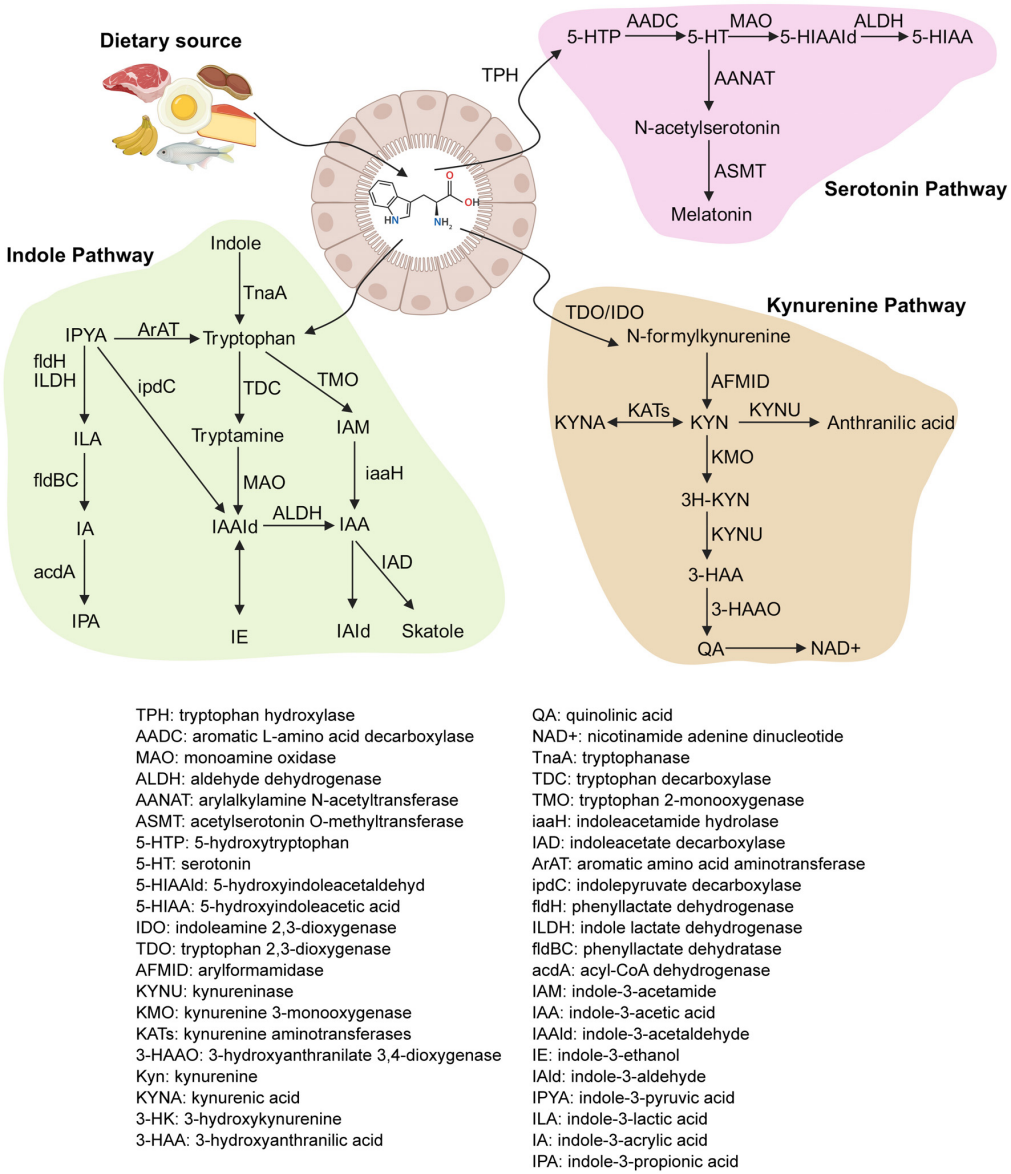
Dietary sources and metabolic pathways of tryptophan (Figure created with BioRender.com).

Mammals cannot synthesize tryptophan *de novo* and must be acquired from dietary sources (for example, red meat, eggs, fish, cruciferous vegetables, and soy products) (18–20). For neonates, breast milk is a major source of tryptophan (21). Following ingestion, mostly dietary tryptophan is absorbed in the small intestine through transporters on intestinal epithelial cells (IECs), including the apical amino acid B0AT1 transporter (*Slc6a19*) and basolateral aromatic amino acid TAT1 transporter (*Slc16a10*) expressed on intestinal epithelial cells (IECs) (22, 23). In the gastrointestinal tract, tryptophan metabolism occurs through three major pathways (Figure 1): the kynurenine pathway [producing kynurenine (KYN) and related metabolites], the serotonin pathway, and the microbial indole pathway.

### 2.1 Kynurenine pathway

Approximately 90%−95% of free tryptophan in mammals is catabolized via the kynurenine pathway (24, 25). This pathway is initiated by two rate-limiting heme enzymes, tryptophan 2,3-dioxygenase (TDO) and indoleamine 2,3-dioxygenase (IDO) (26, 27). The resulting N-formylkynurenine is subsequently deformylated by arylformamidase to yield KYN (28).

TDO and IDO have different evolutionary origins, tissue distributions, and regulatory mechanisms (29). TDO, a homotetrameric enzyme conserved across eukaryotes and prokaryotes (30), is expressed in the liver, where it helps maintain systemic tryptophan homeostasis by regulating circulating levels (31, 32). IDO is found exclusively in eukaryotes as two paralogs: IDO1 appears in most tissues, and IDO2 is limited to kidney, liver, and placenta (33, 34). IDO1 acts within local tissue environments and is highly inducible by proinflammatory stimuli, including cytokines such as interleukins, type I interferons, as well as lipopolysaccharide (LPS), thereby serving as the principal mediator of tryptophan depletion during inflammatory responses (35).

KYN experiences further metabolism via three branches: (1) transamination by KYN aminotransferases to generate kynurenic acid (KYNA); (2) hydrolytic cleavage by kynureninase to produce anthranilic acid; and (3) hydroxylation by kynurenine 3-monooxygenase to yield 3-hydroxykynurenine. Later, 3-hydroxykynurenine is metabolized by KYNU to 3-hydroxyanthranilic acid, which is subsequently transformed by 3-hydroxyanthranilate 3,4-dioxygenase into quinolinic acid. Quinolinic acid is a biosynthetic precursor of nicotinamide adenine dinucleotide, which serves as an essential cofactor in cellular energy metabolism (36).

### 2.2 Serotonin pathway

Tryptophan can be converted to serotonin (5-HT) by tryptophan hydroxylase (TPH) and aromatic amino acid decarboxylase (AADC). TPH exhibits distinct tissue-specific isoforms: TPH2 mediates central 5-HT synthesis in neurons, while TPH1 in enterochromaffin cells accounts for over 90% of peripheral 5-HT production (37). Peripheral 5-HT exerts local effects or is sequestered by platelets for transport to distant organs, including the liver, skeletal muscle, and cardiovascular system (37, 38). The 5-HT reuptake transporter regulates intracellular and extracellular 5-HT concentrations by mediating reuptake and signaling termination (39–41). Monoamine oxidase (MAO) degrades 5-hydroxytryptamine to 5-hydroxyindoleacetaldehyde, which aldehyde dehydrogenase (ALDH) oxidizes to 5-hydroxyindoleacetic acid for urinary excretion. 5-HT in the pineal gland can serves as a precursor for melatonin synthesis, a pathway essential for circadian rhythm regulation (42).

Peripheral 5-HT produces pleiotropic physiological effects by activating specific 5-HT receptors (37). In the gastrointestinal tract, it functions as a signaling molecule that modulates gut motility, vasodilation, and nutrient absorption by transmitting sensory signals to enteric neurons (22, 37). Beyond the gut, 5-HT is important in metabolic homeostasis. It promotes insulin secretion and *de novo* lipogenesis in hepatocytes and white adipose tissue, but suppresses catabolic activity in brown and beige adipose tissues (43–45). Regarding gut-brain communication, mature 5-HT cannot traverse the blood-brain barrier. However, its immediate precursor 5-hydroxytryptophan readily crosses the blood-brain barrier via amino acid transporters and acts as a direct substrate for central 5-HT synthesis (46–48). Thus, through these direct and indirect pathways, 5-HT mediates bidirectional communication between the gut and the central nervous system.

### 2.3 Microbiota-driven indole pathway

A small fraction (4%−6%) of dietary tryptophan escapes small intestinal absorption and reaches the colon, becoming a substrate for bacterial metabolism (11, 49). The bacterial production of indole via tryptophanase (TnaA) was first reported in 1897 (50). TnaA is widely distributed among bacterial species, including *E. coli, Clostridium* spp., and *Bacteroides* spp. (11, 51–53). Multi-omics approaches have progressively elucidated the biosynthetic pathways by which the gut microbiota converts tryptophan into diverse indole derivatives (Table 1).

**Table 1:** Production of indole derivatives by specific gut bacterial species.

Tryptophan metabolite	Bacteria		References
Indole	*Bacteroides thetaiotaomicron*	*Clostridium tetani*	(51–53)
	*Bacteroides ovatus*	*Desulfovibrio vulgaris*	
	*Clostridium bifermentas*	*Escherichia coli*	
	*Clostridium ghoni*	*Escherichia fergusonii*	
	*Clostridium limosum*	*Flavobacteria bacterium*	
Tryptamine	*Clostridium sporogenes*	*Ruminococcus gnavus*	(58, 211)
	*Lactobacillus reuteri*		
Indole-3-acetic acid (IAA)	*Bifidobacterium animalis*	*Clostridium bartlettii*	(51, 54, 55, 105, 212–214)
	*Bacteroides eggerthii*	*Clostridium sporogenes*	
	*Bacteroides fragilis*	*Eubacterium hallii*	
	*Bacteroides ovatus*	*Lactobacillus Salivarius*	
	*Bifidobacterum pseudolongum*	*Lactobacillus reuteri*	
	*Bacteroides thetaiotaomicron*	*Parabacteroides distasonis*	
Indole-3-aldehyde (IAld)	*Bifidobacterium longum*	*Lactobacillus murinus*	(60–62, 215)
	*Lactobacillus acidophilus*	*Lactobacillus reuteri*	
Indole-3-acrylic acid (IA)	*Clostridium sporogenes*	*Peptostreptococcus russellii*	(54, 56, 216)
	*Limosillactobacillus mucosae*	*Peptostreptococcus stomatis*	
	*Peptostreptococcus anaerobius*		
Indole-3-lactic acid (ILA)	*Anaerostipes caccae*	*Bifidobacterium scardovii*	(11, 54, 56, 58, 103, 111, 158, 217–222)
	*Anaerostipes hadrus*	*Clostridium sporogenes*	
	*Akkermansia muciniphila*	*Escherichia coli*	
	*Bacteroides eggerthii*	*Lachnospira eligens*	
	*Bacteroides fragilis*	*Latilactobacillus sakei*	
	*Bacteroides ovatus*	*Lacticaseibacillus paracasei*	
	*Bacteroides thetaiotaomicron*	*Lactobacillus murinus*	
	*Bifidobacterum adolescelentis*	*Lactobacillus johnsonii*	
	*Bifidobacterum bifidum*	*Lactobacillus plantarum*	
	*Bifidobacterium breve*	*Lactobacillus reuteri*	
	*Bifidobacterium kashiwanohense*	*Ligilactobacillus salivarius*	
	*Bifidobacterium longum subsp. infantis*	*Peptostreptococcus anaerobius*	
	*Bifidobacterium longum subsp. longum*		
Indole-3-propionic acid (IPA)	*Clostridium botulinum*	*Enterocloster aldenensi*	(51, 54, 56, 220, 223)
	*Clostridium caloritolerans*	*Peptostreptococcus anaerobius*	
	*Clostridium cadvareris*	*Peptostreptococcus asaccharolyticus*	
	*Clostridium cylindrosporum*	*Peptostreptococcus russellii*	
	*Clostridium paraputrificum*	*Peptostreptococcus stomatis*	
	*Clostridium sporogenes*		
3-methylindole (skatole)	*Actinomyces meyeri*	*Clostridium sporogenes*	(11, 224–226)
	*Butyrivibrio fibrisolvens*	*Eubacterium cylindroides*	
	*Bacteroides thetaiotaomicron*	*Eubacterium rectale*	
	*Clostridium aminophilum*	*Megasphaera elsdenii*	
	*Clostridium bartlettii*	*Megasphaera elsdenii*	
	*Clostridium drakei*	*Megamonas hypermegale*	
	*Clostridium scatologenes*	*Parabacteroides distasonis*	

For instance, Dodd et al. (54) characterized the IPA biosynthetic pathway in *Clostridium sporogenes*. In this pathway, tryptophan is first transaminated by aromatic amino acid aminotransferase (ArAT) to form indole-3-pyruvate (IPYA). In successive steps catalyzed by phenyllactate dehydrogenase (*fldH*), phenyllactate dehydratase (*fldBC*), and acyl-CoA dehydrogenase (acdA), IPYA is converted into ILA, indole-3-acrylic acid (IA), and ultimately IPA. Homologous gene clusters (*fldAIBC*) encoding phenyllactate dehydratase have been identified in the genomes of several anaerobic *Peptostreptococcus* species (e.g., *P. anaerobius, P. russellii*, and *P. stomatis*), as well as in *Clostridium botulinum* and *Clostridium cadaveris*. This is consistent with their capability of producing IPA (54–56). *Lactobacillus* spp. can produce ILA by metabolizing tryptophan via ArAT and indolelactate dehydrogenase (ILDH). *Bifidobacterium* spp. and *Bacteroides* spp. have also been confirmed to produce ILA (11).

Remarkable metabolic diversity was observed in the IAA production, with the gut microbiota employing at least three parallel routes. (1) In the IPYA pathway, ArAT, a widely conserved enzyme, transaminates tryptophan to IPYA, which is then decarboxylated to indole-3-acetaldehyde (IAAld) by indole-3-pyruvate decarboxylase (ipdC) and oxidized to IAA by ALDH (57). (2) In the tryptamine pathway, tryptophan is decarboxylated by tryptophan decarboxylase (TDC) to form tryptamine, which is then converted to IAAld by MAO and ultimately to IAA. Although TDC activity is exceedingly rare in bacteria, metagenomic analyses reveal that at least 10% of human gut metagenomes encode TDC or its homologs (58), particularly *Ruminococcus gnavus* and *C. sporogenes*. (3) In the indole-3-acetamide (IAM) pathway, tryptophan 2-monooxygenase (TMO) catalyzes the conversion of tryptophan into IAM, which is later hydrolyzed to IAA by indoleacetamide hydrolase. Notably, *C. sporogenes, R. gnavus*, and *Bacteroides* spp. have the capacity to produce IAA through distinct routes involving intermediates like IAM and IAAld (59). Downstream of IAA, some *Clostridium* and *Bacteroides* species decarboxylate IAA to generate 3-methylindole (skatole) (51), while IAA can also be converted to IAld. The production of IAld appears to be confined to a small subset of Firmicutes, including *Lactobacillus acidophilus, Lactobacillus murinus*, and *Lactobacillus reuteri* (60–62).

Collectively, these microbial-derived indole compounds function as pivotal signaling molecules in host-microbe communication. By serving as ligands for host receptors, such as AhR and PXR, they activate downstream signaling cascades that modulate intestinal barrier function, immune homeostasis, and systemic metabolism.

### 2.4 Crosstalk and competition among tryptophan metabolic pathways

The three major tryptophan metabolic pathways do not operate separately. They compete for available tryptophan and interact via the gut microbiota and host receptors (63, 64). Shifts in this balance can profoundly influence disease progression and immune homeostasis.

In hypertensive middle-aged females, the depletion of indole-producing bacteria was associated with increased availability of host tryptophan. This is in line with more tryptophan transported to the kynurenine pathway and higher levels of circulating KYN (65). During inflammation, 5-HT derived from enterochromaffin cells seems to affect the activity of the kynurenine pathway (40). Several studies have reported that reduced IDO1 activity is linked to higher 5-HT levels during intestinal inflammation (66–68). As the terminal metabolite of the 5-HT pathway, melatonin also modulates this network via the regulation of IDO1 expression in melanocytes and fibroblasts (69). Melatonin activates the c-Jun N-terminal kinase signaling and promotes the nuclear translocation of forkhead box O1, which then enhances the expression of IDO1 (70). This creates a cross-regulatory loop between both host pathways. In a multiple sclerosis model, IAld derived from microbiota shifted the tryptophan metabolism of mast cells in peripheral lymph nodes toward 5-HT synthesis mediated by TPH1 (71).

However, the complexity of this regulatory network highlights that the main tryptophan metabolic pathways are interdependent. In an atherosclerosis model induced by a high-fat diet (HFD), the depletion of epithelial-specific IDO was related to the reduced synthesis of KYN but the increased accumulation of 5-HT, consistent with the redistribution of substrates, alongside decreased indole metabolites derived from microbiota (63). These observations demonstrate that the perturbation of one pathway can influence the others through substrate competition and regulatory crosstalk, which shifts the overall metabolic balance. Together, these findings underscore that host metabolism is tightly correlated with microbial tryptophan metabolism.

## 3 AhR and PXR mediate microbial tryptophan metabolite signals

AhR is a ligand-activated transcription factor expressed in hepatocytes, IECs, and multiple immune cells, including dendritic cells, type 3 innate lymphoid cells (ILC3s), and Th17 and Th22 cells (72). In the quiescent state, AhR exists in the cytoplasm complexed with chaperone proteins, such as heat shock protein 90 (73). Upon binding to tryptophan-derived metabolites, AhR undergoes a conformational change and nuclear translocation (74). In the nucleus, AhR heterodimerizes with the AhR nuclear translocator (ARNT) and binds to xenobiotic response elements in the promoter regions of target genes, which initiates the transcription of genes such as cytochrome P450 1A1 (*Cyp1a1*) (75). AhR is activated by various tryptophan metabolites, including indole derivatives such as indole, IAA, ILA, and IPA (76). IL-22 is essential for modulating intestinal immunity and inflammation. AhR regulates its secretion via multiple mechanisms. For example, AhR regulates ILC3 proliferation and renewal through Notch signaling, which thus modulates the production of IL-22 (77). AhR activation also promotes IL-22 generation by influencing monocytes and naïve CD4+ T cells (78, 79).

Besides canonical AhR/ARNT-dependent transcription, non-canonical AhR signaling has attracted increasing attentions. AhR serves more as a signaling mediator (80). Ligand activation of AhR promotes the dissociation of the cytosolic AhR complex and releases the soluble tyrosine kinase c-Src (81). Activated c-Src can directly activate the epidermal growth factor receptor (EGFR) on the plasma membrane by phosphorylating its intracellular domain. Additionally, it can sequentially activate protein kinase C (PKC) and sheddases, resulting in ectodomain shedding of cell surface-bound EGFR ligands. Through intracellular phosphorylation or ligand binding, EGFR activation can induce downstream signaling pathways, such as the mitogen-activated protein kinase (MAPK), to affect cellular function (82). For example, it was shown that prenatal exposure to an enriched environment altered maternal gut microbiota composition, increased *Lactobacillus* abundances, and significantly elevated IPA levels in maternal serum and fetal mouse brains. Further *in vivo* and *in vitro* experiments showed that IPA promoted the proliferation and neuronal differentiation of embryonic neural progenitor cells by activating the AhR–Src–Erk1/2 signaling axis (83).

AhR may also contribute to the regulation of cellular metabolism. For example, inhibiting AhR in colon cancer cells reduces the expression of the lipogenic enzyme stearoyl-CoA desaturase 1 (*SCD1*), accompanied by the changes in energy metabolism and fatty acid synthesis, which may inhibit cancer cell proliferation in a cell-specific manner (84). On the other hand, AhR activation can inhibit glycolysis by downregulating molecules associated with glucose transport and glycolysis, such as SLC2A1 and ENO1, thereby reducing glucose uptake and the production of pyruvate and lactate, while promoting SIRT1-dependent differentiation (85).

AhR is the most extensively studied host receptor for tryptophan metabolites, but PXR is another significant receptor in this process (86). PXR belongs to the nuclear receptor superfamily (87). It is mainly expressed in the small intestine and liver, with lower levels detected in the lung, kidney, placenta and ovary (88, 89). Beyond its canonical role in activating drug metabolism and transport genes (e.g., *Cyp3a* and *Oatp1a4*) to clear xenobiotics (90, 91), PXR is a versatile regulator of metabolic and immune homeostasis. It extensively interacts with the nuclear factor kappa-light-chain-enhancer of activated B cells (NF-κB), Toll-like receptors (TLRs), and inflammasomes (92, 93). Moreover, PXR is involved in energy metabolism and apoptosis (94). It has been observed that specific indole derivatives such as indole (95), IAA (96), IAM (97), and particularly IPA (98) are agonists of PXR. IPA acts as a PXR ligand, downregulating TNF-α expression and upregulating tight junction proteins to preserve intestinal barrier integrity (99). Thus, elucidating mechanisms mediated by PXR is essential for comprehensively understanding the host-microbiome tryptophan crosstalk beyond the well-characterized AhR signaling.

## 4 Key biological functions of microbial tryptophan metabolites

**Figure 2:**
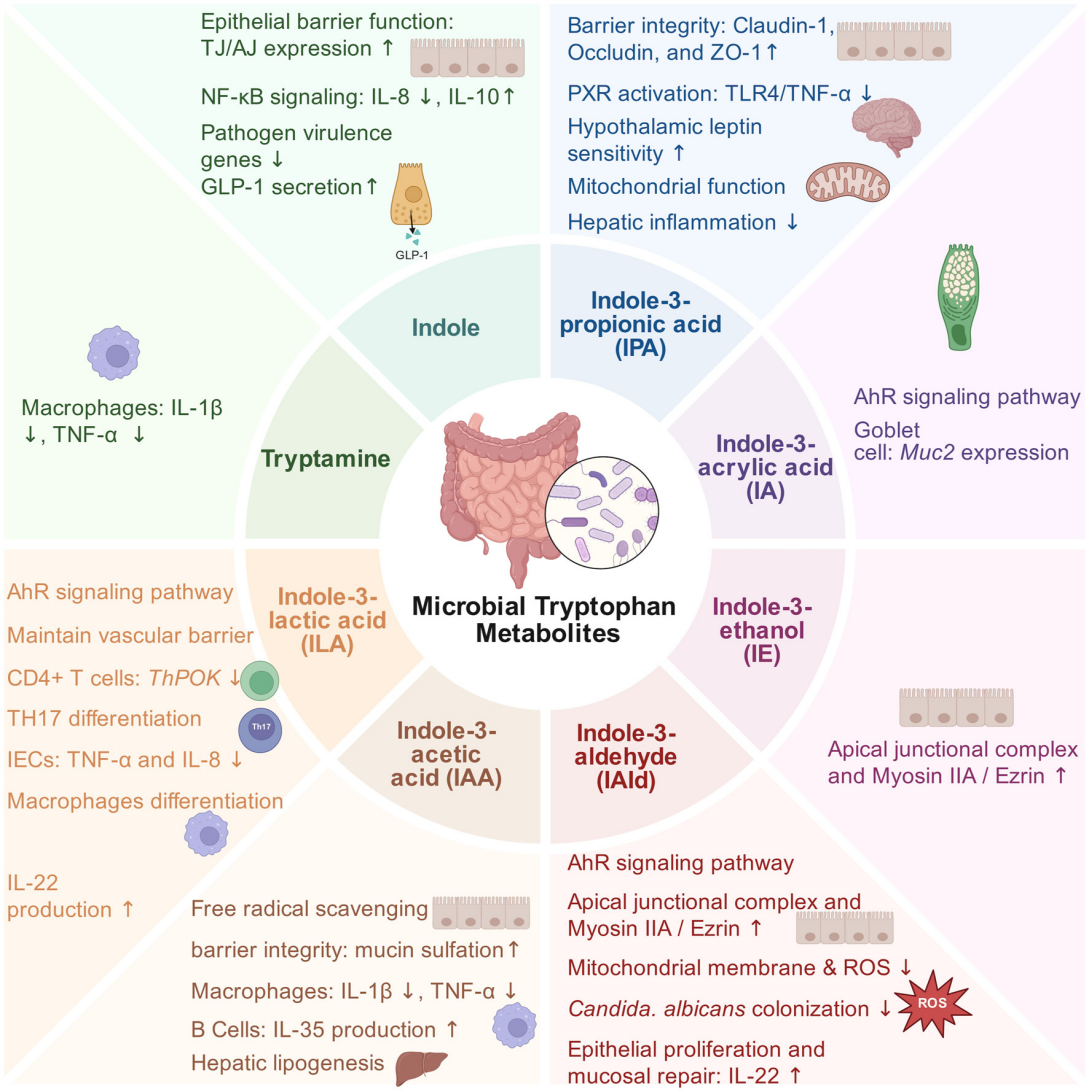
Microbiota-derived tryptophan metabolites mediate host intestinal barrier function, immune homeostasis, and metabolic regulation. AhR, aryl hydrocarbon receptor; AJ, adherens junction; DCs, dendritic cells; IECs, intestinal epithelial cells; NF-κB, Nuclear factor kappa-light-chain-enhancer of activated B cells; PXR, pregnane X receptor; TJ, tight junction (figure created with BioRender.com).

Microbial tryptophan metabolites, including indole, its derivatives (e.g., ILA, IPA, IAA, and IAld) and tryptamine, function as key cross-kingdom signaling molecules regulating host physiology. These metabolites play indispensable roles in reinforcing the intestinal barrier, maintaining immune homeostasis, and modulating host energy metabolism (Figure 2).

### 4.1 Enhancing intestinal physical and chemical barriers

Gut permeability shows a strong association with microbial tryptophan metabolism (100). Dietary tryptophan deficiency has been found to increase intestinal permeability (101). Early *in vivo* studies proved that the oral administration of indole increased the expression of tight junction and adherens junction proteins in the colonic epithelial cells of germ-free mice (102). Consistently, *in vitro* assays indicate that indole at 1 mM induces genes involved in the assembly of tight junction and the formation of actin cytoskeleton, which supports enhanced epithelial barrier integrity (16). Multiple metabolites derived from tryptophan reinforce barrier function through host receptors. As a terminal product of the tryptophan reductive pathway (54, 103), IPA activates PXR to suppress TLR4/TNF-α signaling and increase the expression of junctional genes (99). It also acts as an AhR ligand and enhances the expression of Claudin-1, Occludin and ZO-1 even under LPS-driven inflammatory conditions (104). According to Scott et al. (14), indole-3-ethanol, IPYA, and IAId also regulate the integrity of the apical junctional complex and associated actin-regulatory proteins (including myosin IIA and ezrin) via the AhR pathway. Recent work further indicates that IAA strengthens the intestinal barrier by promoting mucin sulfation through AhR signaling. This depends on upregulation of solute carrier family 35 member B3 and 3′-phosphoadenosine 5′-phosphosulfate synthase 2 (105).

Beyond the epithelial layer, microbial tryptophan metabolites fortify the chemical barrier by promoting mucus production and suppressing pathogen colonization. In a study targeting niche-specific interactions, the mucin-utilizing bacterium *Peptostreptococcus russellii* produces IA near the epithelium, which activates AhR targets (e.g., *Cyp1a1*) and induces the expression of goblet-cell Mucin 2 (56). A similar mucin-promoting effect has been reported for IPA (104). IAld activates AhR to induce the production of IL-22, which supports the stability of commensal communities and enhances colonization resistance against *Candida albicans* (60). Furthermore, high luminal indole concentrations act as a pathogen-sensing signal inhibiting the expression of virulence genes in enteric pathogens, including enterohemorrhagic *E. coli* and *Citrobacter rodentium* (106).

As the third line of defense, the intestinal vascular barrier controls the entry of molecules and microbes into the systemic circulation (107). It reflects the bidirectional relationship between the gut and the liver (108). ILA preserves the integrity of the intestinal vascular barrier through AhR activation in microvascular endothelial cells. This is associated with inactivation of the Nrf2–STAT3 axis and reduced Claudin-2 expression (109).

### 4.2 Regulating host immune homeostasis and anti-inflammatory responses

Indole and its derivatives, including ILA, IAA, and IAld, are endogenous AhR ligands and can modulate the function of immune cells and the production of cytokines. Indole reduces the secretion of pro-inflammatory IL-8 and suppresses the TNF-α-induced activation of NF-κB, while enhancing the expression of anti-inflammatory IL-10 (16). Among these metabolites, ILA plays a vital role in regulating innate and adaptive immunity. ILA generated by *L. reuteri* activates AhR in CD4^+^ T cells. It downregulates the transcription factor ThPOK and reprograms these cells into immunoregulatory CD4^+^CD8αα^+^ double-positive intraepithelial lymphocytes (62). ILA also targets the nuclear receptor RORγt to inhibit Th17 cells differentiation, thereby restricting inflammatory pathology (110). Under the background early-life immune protection, *Bifidobacterium longum subsp. infantis*, which is a dominant strain in breastfed infants, yields ILA suppressing IL-8 surges induced by TNF-α and LPS in IECs (111). As further demonstrated by Meng et al. (112), this protective effect is limited to immature enterocytes. In immature enterocytes, ILA interacts with AhR to inhibit IL-8 transcription induced by IL-1β, which potentially explains the gut health benefits observed in breastfed infants.

These metabolites also regulate innate immune cells. *Bifidobacterium breve*-derived ILA suppresses colitis-associated tumorigenesis by regulating the differentiation of immature colonic macrophages (113). Tryptamine and IAA also suppress inflammatory responses in macrophages, which reduces the production of pro-inflammatory cytokines induced by LPS and fatty acids via AhR-dependent pathways (17).

The AhR-IL-22 axis is an important pathway that links microbial tryptophan metabolites with mucosal immunity. IL-22 is mainly generated by CD3^+^ T cells and ILC3s (114). It drives antimicrobial peptide expression and coordinates epithelial regeneration (115). This axis is modulated by various commensal strains through the production of specific tryptophan metabolites. For instance, *L. reuteri* D8-derived IAld stimulates lamina propria lymphocytes for the secretion of IL-22, which subsequently triggers STAT3 phosphorylation to accelerate mucosal repair (116). Likewise, *L. plantarum*-derived ILA alleviates experimental colitis by activating the AhR-*Cyp1a1*-IL-22-STAT3 cascade (117). IAA from *Bacteroides ovatus* enhances IL-22 production through modulating dendritic cells (118). IAA extends beyond the IL-22 axis. It has also been demonstrated to facilitate immune tolerance by inducing the accumulation of IL-35-secreting regulatory B cells in the intestine (96). Consistent with these protective effects, patients suffering from ulcerative colitis show a reduction in fecal IAA and IAld levels. *Akkermansia muciniphila* supplementation restores these metabolites and relieves inflammation via the upregulation of IL-10 and IL-22 (119).

### 4.3 Mitigating oxidative stress and regulating energy metabolism

Microbial tryptophan metabolites display potent antioxidant activity. IPA and IAA are both able to scavenge free radicals (120, 121). In addition, these metabolites modulate the function of mitochondria as well. IPA stabilizes SIRT1 by suppressing phosphorylation-dependent ubiquitination, enabling SIRT1-driven deacetylation of PGC-1α and promoting its nuclear translocation, thereby activating the SIRT1/PGC-1α pathway and inducing genes involved in mitochondrial biogenesis (122). Consistently, IPA improves the function of mitochondria across disease models. It ameliorates cardiac dysfunction related to metabolic abnormalities (123), protects renal and neural tissues by boosting the content of mitochondrial DNA, promotes biogenesis and mitigates oxidative damage (124, 125). Likewise, IAld supports the integrity of mitochondria by preserving membrane potential and limiting reactive oxygen species through AhR activation, which suppresses the NF-κB–NLRP3 axis (126).

These metabolites also regulate host metabolism. Circulating IPA is linked to the risk of type 2 diabetes and glycaemic control in humans and rodents (127–130). Supplementation with IPA improves glucose metabolism and reduces fasting blood glucose in rat models (129). This metabolic benefit is possibly mediated partly through central mechanisms: IPA functions as a leptin sensitizer. It reaches hypothalamic appetite centers, where it promotes STAT3 phosphorylation and nuclear translocation, thereby enhancing leptin responsiveness and modulating energy intake (131). Indole, another bioactive tryptophan metabolite, stimulates the release of glucagon-like peptide-1 (GLP-1) from intestinal L-cells (132). GLP-1, in turn, boosts insulin secretion and satiety and may help protect against obesity (133, 134).

After absorption, microbial tryptophan metabolites enter the liver through portal circulation and influence inflammatory status and lipid metabolism. Fecal IAA and IPA show a decrease in patients with steatotic liver disease associated with metabolic dysfunction (135). IPA and IAA supplementation dampens hepatic inflammation through the inhibition of NF-κB, the reduction of circulating endotoxin and the restraint of macrophage responses (135). IAA also directly suppresses hepatocyte lipogenesis by downregulating key genes (*SREBF1, SCD1, PPAR*γ, *Gpam* and *Acaca*), which limits fatty acid production (17, 136).

## 5 Nutritional strategies modulating tryptophan metabolism

### 5.1 Dietary patterns

Dietary patterns shape tryptophan metabolism by altering gut microbial community structure and enzymatic activity. Microbial tryptophan catabolism declines when other energy substrates are readily available (137). Multiple animal studies demonstrate that HFD elevates KYN levels (63, 138, 139). In mice, HFD induced IDO1 upregulation, which was accompanied by decreased IAA levels and elevated KYN, indicating a metabolic shift from indole derivative production to KYN generation (140). Consistently, HFD decreases IAA and tryptamine levels in caecal contents and liver (17). In apolipoprotein E-deficient mice, serum IPA is also reduced under HFD and correlates with a lower abundance of indole-producing taxa, including *Clostridium* and *Peptostreptococcus* (141).

In contrast, fiber-rich diets are likely to produce more microbial indoles. Supplementation with wheat bran inhibits activation of the kynurenine pathway while increasing 5-HT and indole metabolites (IPA, IAId and 5-HIAA), potentially through enriching beneficial taxa like *Akkermansia* and *Lactobacillus* (142). The intake of β-glucan, a bioactive dietary fiber, enhances the synthesis of ILA by promoting the proliferation of *Lactobacillus johnsonii* (143). A few human studies have reported that fiber-rich foods (e.g., fruits and vegetables) and fiber intake are positively associated with circulating levels of IPA (127, 128, 144). Moreover, specific fiber combinations yield different indole metabolite profiles. Huang et al. (145) discovered that a combination of pectin and inulin greatly promotes the production of IPA and ILA, whereas pectin alone favors IAA and IAId production. In another study, this group also noticed that high-protein diets do not necessarily elevate levels of indole derivatives. Instead, high-fiber-low-protein diets are favored for the production of IAA, ILA, IAld, and IPA by the colonic microbiota (146).

Collectively, these findings indicate that diets differentially partition tryptophan metabolism. While HFD favors KYN production mediated by IDO1, fiber-enriched diets promote the breakdown of microbial tryptophan into anti-inflammatory indoles through the prebiotic effect. This highlights dietary intervention as a precision nutrition approach for metabolic health.

### 5.2 Probiotic intervention

Probiotics are increasingly used as a way of modulating tryptophan metabolism. This potential is supported by the phylogenetically conserved of key tryptophan-metabolizing enzymes (ArAT, ILDH and acdA) across probiotic genera, especially within *Lactobacillus and Bifidobacterium* (147), suggesting that some strains can directly generate bioactive indole derivatives. Among frequently used probiotics, *L*. *plantarum* extensively exists in fermented foods and shows high acid tolerance and gastrointestinal stress resistance, which are features possibly facilitating intestinal activity (148). Genomic analyses confirm that *L. plantarum* contains genes encoding ArAT, ALDH, and *fldH*, consistent with capacity for IAA and ILA synthesis. Integrative metabolomic and genomic analyses further suggest that high ILA production may be an important probiotic characteristic of *L. plantarum*, with reported benefits associated with AhR activation and attenuation of colitis (117). Furthermore, *L. plantarum* 168 has been shown to produce ILA that specifically targets regulatory CD8^+^ T cells to suppress colorectal tumor growth (149).

In addition, probiotics can modulate the intestinal microenvironment and microbiota composition. This can alter substrate availability and microbial enzyme activity, ultimately shifting tryptophan metabolite profiles. For example, *B. breve* M-16V supplementation has been demonstrated to act with the resident microbiota synergistically and expand taxa associated with tryptophan metabolism, such as *Lactobacillus, Clostridium, Blautia*, and *Roseburia*. Meanwhile, treatment with *B. breve* M-16V reduces the expression of jejunal TDO in mice. This finding suggests that probiotic interventions are likely to influence microbial pathways, the utilization of tryptophan and the expression of host metabolic enzymes, which promotes the production of IPA, IAA and other indole derivatives (150). Additionally, supplementation with *Bifidobacterium longum* CCFM1029 altered gut microbial β-diversity and maintained the abundance of *Lachnospiraceae*, which was accompanied by modulation of tryptophan metabolism and activation of AhR-mediated immune responses. Current evidence also highlights the critical role of multi-species coordination and metabolic cross-feeding. Specifically, *L. reuteri* I5007 converts tryptophan to ILA. ILA is a metabolite that subsequently promotes the colonization of tryptophan-catabolizing commensals (e.g., *Clostridium* spp.) and upregulates bacterial acdA and ILDH expression *in vivo*, which thereby amplifies the production of IPA and IAA (151). Consistent with this, *C. sporogenes* is a potential probiotic candidate. It produces IPA working synergistically with SCFAs and branched-chain fatty acids to promote the production of IL-22 and enhance the activity of Treg cells, which thereby suppresses intestinal inflammation (152). These findings suggest that probiotic-associated physiological benefits generally arise from interactions among microbial community members rather than from the isolated action of a single metabolite or strain.

Next-generation probiotics also show promise. *Bacteroides xylanisolvens* produces IAA and ILA (153, 154). *A. muciniphila* degrades intestinal mucins and produces SCFAs (155), which stimulate TPH1 expression in enteroendocrine cells and promote 5-HT secretion (156, 157). In colitis models, *A. muciniphila* supplementation restores intestinal IAA and improves barrier function (119). Moreover, untargeted metabolomics has detected ILA in *A. muciniphila* culture supernatants and, for the first time, suggested a potential role for ILA in bile-acid metabolic regulation (158). However, the specific genes responsible for ILA biosynthesis in *A. muciniphila* remain insufficiently characterized and require further research.

## 6 Roles of tryptophan and its microbial metabolites in swine health and production

Tryptophan serves not only as a substrate for protein synthesis, but also as a signaling molecule regulating intestinal homeostasis, immune function and metabolic activity in animals. Researchers used a catheterization model in healthy and conscious pigs to assess the interorgan flux of selected microbial metabolites. Seven indole derivatives were identified in pig plasma, and IAA and IA showed a net release from the intestine and were significantly taken up by the liver (159). In addition, IPA and ILA have been detected in pig serum or plasma under specific dietary interventions (160, 161). Collectively, these *in vivo* studies provided direct or indirect evidences that some indole derivatives in pigs can enter systemic circulation. Therefore, a growing number of studies are no longer limited to the nutritional value of tryptophan itself, but rather focusing on the potential of its microbial metabolites in improving the production performance and gut health of pigs, which is of particular importance in early life stages, particularly, the time around weaning. Key studies supporting these beneficial effects, along with the metabolomics methodologies used, are outlined in Table 2.

**Table 2:** Effects of tryptophan and its microbial metabolites on swine health and production.

Study	Intervention	Metabolomics approach	Alterations in tryptophan metabolites	Key findings	References
Comparison between healthy and diarrheal piglets	/	Feces; LC-MS/MS	↓ IAld, 3-indolebutyric acid, etc.	Tryptophan metabolism was reduced in diarrheal piglets	(167)
Weaned piglets	IAId (100 mg/kg)	/	/	Improved intestinal barrier function; promoted ISC expansion	(167)
Piglets (LPS-induced)	FMT	Colonic contents; LC-MS/MS	↑ IAA	Restored epithelial integrity; mitigated inflammatory responses	(169)
Weaned piglets	Tryptophan (0.35%)	Serum; HPLC	↑Tryptophan	Reduced diarrhea incidence and serum IL-6 levels	(171)
Weaned piglets	*Clostridium butyricum*	Feces; LC-MS	↑ Indole-3-carboxylic acid	Optimized Firmicutes/Bacteroidetes ratio; improved piglet health	(172)
Piglets (DSS-induced colitis)	*Lactobacillus salivarius* MZ27	Colonic contents; LS-MS	↑ ILA	Improved intestinal barrier function	(173)
Piglets	Pectin (5%)	Jejunum mucosa; LS-MS	↑ IPA, IAA, Tryptamine	Enriched *Lactococcus* and *Enterococcus*; activated AhR/IL-22/STAT3 axis	(174)
Weaned piglets	garlic-derived exosome-like nanoparticles	Colonic content; LC-MS	↑ IPA	Mitigated stress-related intestinal mucosal inflammation; enhances mucin production	(176)
Weaning piglets	Tryptophan (0.2% or 0.4%)	Cecal and colonic contents; HPLC	↑ IAA, indole	Increased ADFI and ADG	(179)
Piglets	IPA (0.01%)	Plasma and intestinal contents; IPA and tryptamine ELISA kit	↑ plasma IPA; ↓ intestinal tryptamine	Promoted muscle growth and glycolytic fiber formation	(160)
Low birthweight piglets	Tryptophan (0.4% or 0.8%)	/	/	Inhibited hepatic lipogenesis and gluconeogenesis; enhanced glycolysis and lipolysis.	(186)
Finishing pigs	Tryptophan (0.78%)	/	/	Suppressed FXR signaling; improved lipid metabolism	(187)
Weaned piglets	*Lactobacillus plantarum* JL01	Cecum contents; LC-MS	↑ IAA	Modulated microbial composition; improved fat digestion and absorption	(188)
Growing pigs	Time-restricted feeding	Colonic digesta and serum; LC-MS	↑ ILA (colon and serum)	Promoted *Lactobacillus* colonization; reduced hepatic lipid deposition	(161)

ISC, intestinal stem cells; ADFI, average daily feed intake; ADG, average daily gain; FXR, farnesoid X receptor; LPS, lipopolysaccharide; DSS, dextran sulfate sodium; FMT, fecal microbiota transplantation; LC–MS/MS, liquid chromatography–tandem mass spectrometry; HPLC, high-performance liquid chromatography. ↑ indicates increased; ↓ indicates decreased.

### 6.1 Regulating intestinal health and barrier function in swine

Gut health is a multidimensional concept that encompasses metabolic homeostasis, digestion and absorption, microbial ecosystem balance, and immune barrier integrity (162, 163). The significant changes in the diet and environment during the weaning period of piglets trigger serious stress responses, damage intestinal epithelial structure, and cause diarrhea (164). Growing evidence shows that diarrhea in piglets is closely linked to tryptophan metabolism disturbances (165). Piglets have increased tryptophan demand during immune challenges like weaning stress and LPS exposure (166). The tryptophan metabolism of diarrhea piglets is suppressed compared with that of healthy controls. It is accompanied by a reduction in indole derivatives (IAId, 2-indole carboxylic acid, indole-3-carboxylic acid and 3-indolebutyric acid) (167). Multiple studies support this finding (168–170). The transplantation of the fecal microbiota restores the biosynthetic capacity of indole by gut microbiome modulation and greatly elevates levels of colonic IAA. This metabolic shift activates AhR signaling and upregulates IL-22, which alleviates the disruption of intestinal epithelial integrity and mitigates inflammatory responses induced by LPS (169).

Rao et al. (171) demonstrated that 0.35% tryptophan supplementation in the diet of weaned piglets reduces the incidence of diarrhea and serum IL-6 levels. Microbiome analysis revealed that supplementation with tryptophan increases the abundance of *Lactobacillus* and *Ruminococcaceae* while reducing the abundance of *Turicibacter, Desulfovibrio* and other potential pathogens. *Clostridium butyricum*, a feed additive, enhances indole-3-carboxylic acid levels in weaned piglets and optimizes the ratio of Firmicutes to Bacteroidetes. This suggests that *Clostridium* promotes the health of piglets via the regulation of tryptophan metabolism (172). Zheng et al. (173) isolated *Lactobacillus salivarius* MZ27 from Min pigs and confirmed that it ameliorates dextran sulfate sodium-induced colitis by promoting ILA generation and activating AhR signaling. Supplementation with 5% pectin in the diet changed jejunal microbial composition (increasing *Enterococcus* and *Lactococcus*) and increased levels of tryptophan metabolites (including tryptamine, IPA and IAA). These metabolites subsequently activated the AhR/IL-22/STAT3 signaling pathway, which hence reduced pro-inflammatory cytokines and fortified intestinal barrier integrity (174).

Dietary supplementation with 50 and 100 mg/kg IPA reduces diarrhea incidence, alleviates intestinal inflammation induced by LPS, and increases the expression of tight junction proteins in weaned piglets. However, high doses of IPA (600 mg/kg) may induce pro-inflammatory responses, which lays emphasis on the importance of optimizing doses (175). Dietary IAld improves barrier function and intestinal development by promoting the proliferation of intestinal stem cells in weaned piglets (167). Oral administration of garlic-derived exosome-like nanoparticles (50 mg/kg body weight) mitigates stress-related intestinal mucosal inflammation and enhances mucin production in weaned piglets, likely by enriching *Lactobacillus* (specifically *L. reuteri*) and inducing production of the anti-inflammatory metabolite IPA (176). Overall, these findings indicate that nutritional interventions aimed at tryptophan and its metabolites may provide novel strategies for preventing and alleviating diarrhea in piglets.

### 6.2 Enhancing growth performance and regulating metabolism and stress responses in swine

Tryptophan is an essential amino acid. Sufficient dietary supply is essential to the optimization of growth performance and health in swine, especially young piglets (177, 178). Supplementation with tryptophan (0.2%−0.4%) elevates average daily gain and average daily feed intake while increasing the diversity of the gut microbiota (179). In low-protein diets, supplementation with tryptophan and N-acetylglutamic acid together improves the ratio of feed-to-gain and enhances growth performance, with substantially greater average daily gain and feed intake (180).

Skeletal muscle accounts for approximately 50% of pig body weight (181). It is central to meat quality and growth performance in swine production (160). Tryptophan and its metabolites contribute to the growth and development of animals, particularly through the regulation of muscle development (182, 183). Early trials suggested that high dietary tryptophan levels (a body weight of 1.3–2.0 g/kg) promote growth and feed efficiency, potentially by regulating ribosomal activity and protein synthesis in muscle cells (184). A diet high in tryptophan (0.35%) increases the proportion of fast-twitch muscle fiber in weaned piglets via a circular RNA network targeting miR-34c and miR-182 (183). Supplementation with the IPA promotes the growth of muscles and the formation of glycolytic fiber by improving insulin sensitivity and activating the PI3K-Akt-mTOR pathway (160). In murine models, *C. sporogenes* and its metabolite IPA repair muscle atrophy induced by antibiotics (185).

Tryptophan and its metabolites also regulate lipid metabolism in swine. Dietary tryptophan supplementation improves the metabolic pattern of low birthweight piglets, which inhibits hepatic lipogenesis and gluconeogenesis while enhancing lipolysis and glycolysis (186). In finishing pigs, 0.78% tryptophan suppresses intestinal farnesoid X receptor signaling and increases the synthesis of hepatic bile acid, which may be beneficial for lipid metabolism (187). Probiotic *L. plantarum* JL01 enhances IAA production *in vivo* and modulates lipid metabolism in weaned piglets (188). Similarly, Li et al. (161) demonstrated that time-restricted feeding promotes the colonization of *Lactobacillus* and the generation of ILA in growing pigs; ILA subsequently upregulates PPARα and CPT1A via the AhR pathway, reducing serum and hepatic lipid deposition. It has been proven that ILA derived from *Lactobacillus* elevates GLP-1 levels through promoting intestinal stem cell differentiation into enteroendocrine cells (189). This increase in GLP-1 may reduce the accumulation of subcutaneous and hepatic fats through the regulation of appetite and energy expenditure, which thereby improves carcass traits (190).

In pigs, management-associated stressors, such as heat stress, transport, weaning, farrowing, slaughter and unsuitable housing conditions can compromise animal welfare and reduce production performance (191). Recent studies in mice suggested that indole derived from the gut microbiota might play a role in regulating the gut-brain axis (192). It is believed that the amygdala plays an essential role in regulating stress responses and anxiety-related behaviors (193). Male GF mice exhibit anxiety-related behaviors and heightened fear responses, which may be associated with hyperexcitability of excitatory neurons in the basolateral amygdala. Indole treatment reduced behaviors associated with anxiety and attenuated the hyperexcitability of basolateral amygdala neurons in GF mice. This suggests that indole may modulate brain circuits involved in emotional regulation (194). Although current studies have limited to mouse models, these findings raise the possibility that microbial tryptophan metabolites may also contribute to stress resilience and welfare in pigs.

## 7 Skatole: a potentially adverse metabolite of microbial tryptophan metabolism in swine

Extensive literatures have emphasized the beneficial roles of microbial tryptophan metabolites in maintaining gut barrier integrity and immune homeostasis. Nevertheless, it is critical to recognize the adverse effects of skatole, particularly in swine production. Skatole is produced by intestinal bacteria through the decarboxylation of IAA (51). A portion of the skatole produced in the gut is excreted in the feces, while the rest is transported to the liver via the portal vein and metabolized by cytochrome P450 (CYP) isoenzymes (195). CYP isoenzymes, including CYP1A, CYP2A and CYP2E1, are responsible for skatole degradation (196). Because of its lipophilic nature, skatole that is not degraded in the liver easily deposits in the adipose tissue of pigs (197). Sex hormone levels in pigs are important regulators of cytochrome P450 enzyme activity (137, 198). Androstenone secreted by testicular interstitial cells inhibits the activity of CYP (198). Compared with uncastrated boars, sows exhibit higher CYP1A2, CYP2A, and CYP2E1 activity in their livers (199). Furthermore, the expression and activity of CYP2E1, CYP2A and CYP1A genes in the livers of boars tend to increase after castration or immunocastration (198). Therefore, the effects of skatole in pigs are primarily reflected in the meat quality of boars. Together with androstenone (200), skatole is considered a principal cause of boar taint.

Due to the close association between skatole formation and the gut microbiota, feeding management and dietary regulation in livestock production may help reduce the formation of skatole from the source. Studies have shown that the supplementation with chicory root or pure inulin affects skatole levels in pig feces, blood, and adipose tissue (201, 202). Okrouhlá et al. (203) reported that dietary supplementation with *Helianthus tuberosus* reduced skatole concentrations in adipose tissue, possibly due to a decrease in the abundance of proteolytic bacteria in the gastrointestinal tract, and did not exert significant adverse effects on growth performance or carcass traits in pigs. The diet supplemented with 6% of mulberry leaves significantly reduced the levels of skatole in the feces, serum, and backfat of finishing pigs through multiple mechanisms. Specifically, the mulberry leaf-supplemented group altered the composition of the gut microbiota by reducing the abundance of the known skatole-producing bacteria *Megasphaera* and *Olsenella*, and increasing the *Ruminococcus 1, Anaeroplasma*, and *Butyrivibrio*. This intervention also significantly lowered fecal IAA levels, thereby inhibiting the key precursor for skatole synthesis. Finally, mulberry leaf treatment significantly upregulated CYP1A1 expression in pig livers, which accelerates the degradation of skatole (204). Similarly, the plant polyphenol magnolol was shown to reduce skatole levels in the pig colon and feces by modulating IPYA metabolism; specifically, magnolol can bind to ipdC in *Desulfovibrio*, thereby lowering IAA production and limiting skatole formation (205).

## 8 Conclusions and perspectives

This review discusses how tryptophan metabolism, specifically the microbiota-driven indole pathway, influences gut health, immune homeostasis, and metabolic function. As ligands of AhR and PXR, microbial tryptophan metabolites reinforce the intestinal barrier and metabolic balance, contributing to improved growth and immune function in livestock. However, not all indole metabolites exert beneficial effects. In pigs, IPA shows dose-dependent effects. High doses of IPA may induce a pro-inflammatory response, while skatole is closely associated with boar taint and reduced meat quality. Therefore, future researches should aim to elucidate how nutritional interventions can influence the tryptophan metabolism of pig gut microbiota to coordinate the production of tryptophan metabolites. For instance, a recent study has revealed that dietary fiber can affect the metabolism of tryptophan through microbial metabolic interactions, thereby reducing certain potentially detrimental indole derivatives (103).

Although tryptophan metabolism is usually attributable to genera including *Clostridium, Bacteroides, Escherichia*, and *Lactobacillus*, etc., the metabolic potential of the gut microbial community far exceeds what is suggested by taxonomy alone. Identifying the specific producers of different indole derivatives is important for designing targeted probiotics and precision metabolic therapies. This is particularly true because some metabolite outputs arise from the cooperation of interspecies rather than isolated strains. For instance, *L. johnsonii* and *C. sporogenes* support the production of IPA through cooperation (206). Competitive interactions can shift tryptophan substrate partitioning within microbial communities. In a defined tripartite community, the fiber-degrading *Bacteroides thetaiotaomicron* promotes *E. coli* growth through monosaccharide cross-feeding, thereby suppressing indole formation via catabolite repression and shifting tryptophan partitioning toward ILA and IPA production by *C. sporogenes* (103). Current knowledge may scratch only the surface of this metabolic potential. Untargeted metabolomics, in conjunction with MS/MS-based structural annotation and validation with authentic standards, is essential to identifying novel indole derivatives and linking them to immune-relevant phenotypes. However, metabolite annotation continues to be a major challenge in untargeted metabolome analysis. Relying solely on accurate mass, database matching, or retention times remains insufficient to rule out the risk of misannotation caused by structural isomers, adducts, in-source fragmentation, and co-eluting compounds (207). High-confidence identification of metabolites usually still depends on the integrated matching of retention times, precursor ions, and MS/MS spectra with authentic standards under the same analytical conditions (208, 209).

Microbial tryptophan metabolites maintain immune and tissue homeostasis through AhR and PXR signaling. Their detection in circulation supports the possibility of systemic effects beyond the gut (54, 159). However, the circulating concentrations of indole derivatives in pigs remain poorly defined. Accordingly, an important priority is to use metabolomics for generating quantitative reference ranges and mapping the spatiotemporal distribution of main indole derivatives across intestinal segments, blood and peripheral organs in humans and pigs. It should also be noted that many current mechanistic evidences regarding tryptophan metabolites comes primarily from mouse models and *in vitro* systems. Findings from mice are better suited as mechanistic hypotheses, while the tissue distribution, physiological concentrations, and functional effects of key metabolites still need to be further validated in pigs. Despite the ability of diets and probiotics to modulate tryptophan metabolism, it is still necessary to fully elucidate the precise molecular mechanisms and long-term consequences of these interventions. Addressing these gaps requires large, well-phenotyped cohorts and integrative validation using multi-omics datasets such as metagenomics, targeted metabolomics and spatial transcriptomics (210).

To conclude, tryptophan metabolism is an indispensable interface between diet, host immunity and gut microbes. In swine production, interventions aimed at this axis via microbiome modulation and precision nutrition could improve animal performance while decreasing dependence on antibiotics, which supports more sustainable farming. In addition to agriculture, considering the status of pigs as an important biomedical model, a deep understanding of tryptophan metabolism in swine has great translational value, which informs strategies for optimizing metabolic function and gut health in humans.
